# Investigating Infrared Radiation on Peeling and Roasting Chestnut Seeds (*Castanea sativa Mill*.) and Its Effect on the Physical, Chemical, and Sensory Characteristics of the Product

**DOI:** 10.1002/fsn3.70061

**Published:** 2025-02-27

**Authors:** Shima Ezzati, Majid Javanmard dakheli, Hamed Ahari, Hossein Ahmadi Chenarbon, Gholamhassan Asadi

**Affiliations:** ^1^ Department of Food Science and Technology, Science and Research Branch Islamic Azad University Tehran Iran; ^2^ Department of Chemical Technologies Iranian Research Organization for Science and Technology (IROST) Tehran Iran; ^3^ Department of Agronomy, College of Agriculture, Varamin ‐ Pishva Branch Islamic Azad University Varamin Iran

**Keywords:** chestnut, infrared radiation, optimization, peeling, roasting

## Abstract

The application of infrared radiation has proven effective for roasting, peeling, and inactivating microbes in food, leading to its increased use. This study explores its use for chestnut processing and its effects on storage. Response surface methodology (RSM) optimized conditions for both infrared roasting and peeling: Radiation power (490.5 W), duration (21.42 min), distance (5.55 cm), and initial moisture content (13.77% and 2.72%). Under these conditions, the physicochemical properties, antioxidant content, color, mold count, microstructure, and sensory characteristics of chestnut samples were evaluated during 60 days of storage in polyethylene bags. Storage generally increased hardness and decreased moisture across all chestnut samples, but those processed with infrared radiation exhibited superior outcomes. Hot air roasting yielded the highest phenolic content. Initial color analysis showed no significant difference between IR and control groups, except for manually peeled samples exhibiting lower lightness (L* index) and browning index. Notably, on day 30, IR‐roasted chestnuts had the lowest mold count, while hand‐peeled samples had the highest. Finally, after 60 days, sensory evaluation revealed the lowest overall acceptance scores for hand‐peeled chestnuts, while IR‐roasted samples received the highest ratings. These findings demonstrate that IR technology for both roasting and peeling significantly improves the quality and sensory attributes of chestnuts during storage, suggesting its potential for industrial application.

## Graphical abstract

This study investigates the application of infrared radiation (IR) for processing chestnuts, focusing on optimizing roasting and peeling conditions using response surface methodology. The optimized IR‐treated chestnuts displayed improved physicochemical properties, enhanced antioxidant content, and superior sensory attributes over 60 days of storage. The findings suggest that IR technology significantly enhances the quality and storage stability of chestnuts, offering promising industrial applications.

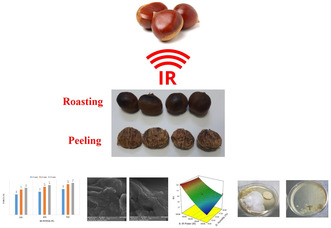

## 1 Introduction

Driven by the search for innovative and sustainable methods, the food science community has increasingly focused on advanced food processing techniques in recent years. Infrared radiation, particularly promising for nut processing due to its nutritional value and culinary applications, has emerged as a frontrunner among the explored methods (Bagheri 2020).

Chestnut seeds, derived from the *Castanea sativa Mill* tree, commonly known as the sweet chestnut or European chestnut, hold a significant place in culinary traditions and ecosystems across the globe. Characterized by their rich, sweet flavor and soft, crumbly texture when cooked, these seeds are not only a source of delicious food but also carry substantial nutritional value. Chestnuts, unlike other nuts, possess a unique composition, which includes a high moisture content and a low‐fat content, making them akin to grains in some respects (Lixia et al. 2015; Mustafa et al. 2021). Traditional methods of processing chestnuts, specifically peeling and roasting, often involve labor‐intensive techniques that can lead to significant product loss and inconsistency in the quality of the end product (Mustafa et al. 2021).

Roasting chestnuts plays a critical role in nut product quality. Numerous physical, chemical, and sensory transformations occur during this process, not only enhancing taste, color, and texture but also boosting food safety by eliminating harmful microbes and enzymes (Tu et al. 2021). Temperature, time, initial moisture content, and airflow rate are critical factors for controlling both roasting quality and efficiency. Inadequate roasting parameters can lead to compromised product quality, reduced shelf life, and a loss of desirable characteristics. Roasting triggers significant changes, including nonenzymatic browning reactions, alterations in carbohydrates, proteins, and fats, as well as the degradation of aflatoxins (Chandrasekara and Shahidi 2011; Schlörmann et al. 2015).

Infrared radiation emerges as a novel approach, potentially providing a more uniform heating process. This could not only accelerate peeling and roasting but also elevate the product's overall quality. Unlike traditional methods, infrared radiation penetrates food materials, directly heating them from the inside out. (Amiri Chayjan et al. 2014). This internal heating method fosters more uniform cooking and drying, especially valuable for chestnuts where delicate flavors and textures demand careful processing. Furthermore, the impact of infrared radiation on physical characteristics like chestnut texture and color remains paramount. After all, these attributes significantly influence consumer acceptance and marketability (Buthelezi et al. 2019). Similarly, changes in the chemical composition of chestnuts, including alterations in nutritional value and the formation of flavor compounds during roasting, are critical factors when assessing the suitability of infrared processing (Pan 2020).

Infrared peeling has been successfully applied to various nuts and fruits, including jujube (Wang et al. 2016), hazelnut (Eskandari et al. 2018), pear (Shen et al. 2020), ginger (Kate and Sutar 2020), and tomato (Qu et al. 2022). Moreover, Belviso et al. (2017) reported that roasting hazelnuts at 120°C for 40 min using a hot air system enhances oxidative stability, with optimal antioxidant activity maintained through 6 months of storage at 4°C (Belviso et al. 2017). Different effects of infrared roasting on properties such as aflatoxin decontamination of hazelnuts (Siciliano et al. 2017), hazelnut allergenicity (Lamberti et al. 2021), textural, color, and sensory attributes of peanut kernels (Bagheri et al. 2019), physicochemical properties of wild almond (Mokhtari and Ziaiifar 2018) and functional and digestibility of walnut kernel (Zhao et al. 2024) were also reported. This paper investigates the use of infrared radiation for peeling and roasting chestnut seeds. Through a comprehensive analysis of how infrared radiation affects the physical, chemical, and sensory characteristics of chestnut seeds, this study aims to provide valuable insights that could significantly improve chestnut processing methods.

## 2 Materials and Methods

### 2.1 Materials

In this study, chestnut samples were prepared from a local market in Tehran and, after sizing, samples of uniform dimensions were selected for experimentation. All other chemicals used in this study were of analytical grade and purchased from chemical suppliers.

### 2.2 Peeling Samples Using the Traditional Method

A total of 500 g of chestnuts was prepared for the study. Five hundred grams served as control samples and were manually peeled with a knife (Fernando et al. 2014).

### 2.3 Peeling With Infrared

An infrared roaster was used for the peeling process, following a Box–Behnken experimental design. Chestnut samples (250 g) were exposed to infrared radiation at varying power levels (300, 400, and 500 W), durations (10, 20, and 30 min), and distances from the source (5, 10, and 15 cm). The experiment was conducted in triplicate with samples having initial moisture contents of 10%, 15%, and 20%. To ensure uniform heat distribution, the samples were continuously rotated at 1 rpm on a rotating plate within a custom metal holder (Kate and Sutar 2020; Wang et al. 2016).

### 2.4 Roasting With Hot Air

To ensure stable and uniform conditions at the beginning of roasting, the electric oven with air circulation was preheated to 200°C for 15 min before each experiment. Then, 250 g of chestnuts, peeled with a knife and having an initial moisture content of 10%, was roasted for 10, 20, and 30 min. The roasted samples were stored in zippered polypropylene packaging until the time of testing after reaching room temperature (Pan 2020).

### 2.5 Infrared Roasting Process

An infrared roaster was employed for the roasting process, following a Box–Behnken design. Chestnut samples (250 g) were subjected to infrared radiation at varying power levels (300, 400, and 500 W), durations (10, 20, and 30 min), and source distances (5, 10, and 15 cm). The experiment was conducted in triplicate with samples having initial moisture contents of 10%, 15%, and 20%. To ensure uniform heating throughout, the samples were continuously rotated at 1 rpm on a rotating plate within a custom metal holder (Bagheri et al. 2019; Fernando et al. 2014).

### 2.6 Optimal Selection of Peeling and Roasting With Infrared

Design Expert software was used for treatment coding with a composite mixture design, and the response surface methodology with a central composite design was employed for process optimization.

### 2.7 Analyses of Peeled and Roasted Chestnut Seeds

All samples will be stored for 60 days in zippered polyethylene bags, and tests will be conducted on the samples at three time intervals (days 0, 30, and 60).

#### 2.7.1 Measuring the Final Moisture Content

The final moisture content of the samples for each of the peeling and roasting methods was calculated. This involved roasting peanut kernels until they reached a constant weight according to the treatments defined for each method. Subsequently, the moisture content of the roasted sample was measured in three replicates using an oven, according to the formulas below, and reported based on dry weight (Wani et al. 2017).

$$\mathrm{MCr}\;\left(\%\mathrm{wb}\right)=M_{0}r-\mathrm{Mr}/M_{o}r\times 100$$

$$\mathrm{Mdr}\;\left(\%\mathrm{wb}\right)=\mathrm{MCr}\times 100/100-\mathrm{MCr}$$

where *M*0*r* is the initial weight of the roasted chestnut seed, *Mr* is the final weight of the roasted kernel, *MCr* is the moisture content based on the wet basis, and *Mdr* is the moisture content based on the dry basis.

#### 2.7.2 Measuring the Texture

Texture analysis was performed using a Texture Analyzer (TA.XT Plus, Stable Micro Systems, UK) with a compression test. A cylindrical probe (diameter 25 mm, P/2 N) penetrated the samples at a speed of 5 mm/min to determine textural properties (Mohammadi Moghaddam et al. 2016).

#### 2.7.3 Scanning Electron Microscopy

The microstructure of the samples was analyzed using an electron microscope (Philips XL/30) following the method described by Schmiele (2015). In this method, samples were mounted on a carbon strip, gold‐coated, and placed on a special stand for observation at magnifications of ×100 and ×500 with an acceleration voltage of 10 kV and a beam current of 50 pA (Schmiele 2015).

#### 2.7.4 Measuring Antioxidant Activity

The antioxidant activity of samples is assessed using the 2,2‐diphenyl‐1‐picrylhydrazyl (DPPH) free radical scavenging method. This method measures the capacity of samples to neutralize free radicals by monitoring the decrease in absorbance of a DPPH solution at 517 nm after mixing with sample solutions of varying concentrations. Lower absorbance indicates greater antioxidant efficacy. BHT is used as a standard for comparison. The DPPH radical scavenging activity (%) is then calculated using the following formula (Wani et al. 2017):

DPPH free radical scavenging activity=Absorption of control/Absorption of sample

#### 2.7.5 Color Characteristics

To examine and analyze the color characteristics of roasted and peeled seeds, a Hunter Lab colorimeter was used. The parameters L* (lightness), a* (red‐green), b* (yellow‐blue), ∆E (total color difference), Hue angle, Chroma index, and the browning index were investigated (Proietti et al. 2021).

#### 2.7.6 Nonenzymatic Browning Index

The degree of nonenzymatic browning was determined using a spectrophotometer and an absorbance method. Two grams of powdered chestnut seeds was homogenized with 40 mL of distilled water. The homogenate was then mixed with 10 mL of 10% trichloroacetic acid (TCA) solution and incubated at 30°C for 2 h. The mixture was filtered using filter paper, and 1 mL of the filtrate was diluted threefold. The diluted filtrate's absorbance was measured at 420 nm using a spectrophotometer (Chung et al. 2014).

#### 2.7.7 Total Phenolic Compound Content

The study utilized the Folin–Ciocalteu colorimetric method to quantify the total phenolic content in samples, with results expressed in gallic acid equivalents. This involves inducing a color change through the reduction of phosphotungstomolybdic acid by phenolic compounds in an alkaline medium, with the intensity of the resulting blue color measured spectrophotometrically at 760 nm. The process included the extraction of phenolic compounds using a methanol–water solvent, followed by clarification and analysis. The content of phenolic compounds was then calculated based on a calibration curve, providing an estimate of antioxidant capacity in the samples.

#### 2.7.8 Sensory Analysis

For the sensory evaluation of chestnut samples, 20 trained assessors participated. Using a 5‐point hedonic scale, they assessed various characteristics, including color, aroma, roast level, taste and flavor, firmness, and overall acceptance (Porretta 2019).

#### 2.7.9 Mold and Yeast Count

To count mold and yeast in the samples, initially, 5 g of the sample was mixed with 45 g of Ringer's diluent solution to create a suspension, which was then diluted to 0.01. Subsequently, two plates containing DG18 agar medium were inoculated with 0.1 mL each of the first and second dilutions of the sample using a sterile pipette; these were then incubated at 25°C for 3–5 days.

### 2.8 Data Analysis

The data were analyzed in the framework of a completely randomized design with three replications. Analysis was conducted using SPSS software version 24, and mean comparisons were made using Duncan's multiple range test. Excel software was utilized for plotting graphs. The error level for all traits was set at 0.05.

## 3 Results and Discussion

### 3.1 Optimization of Peeling Conditions Using Infrared Radiation

**FIGURE 1:**
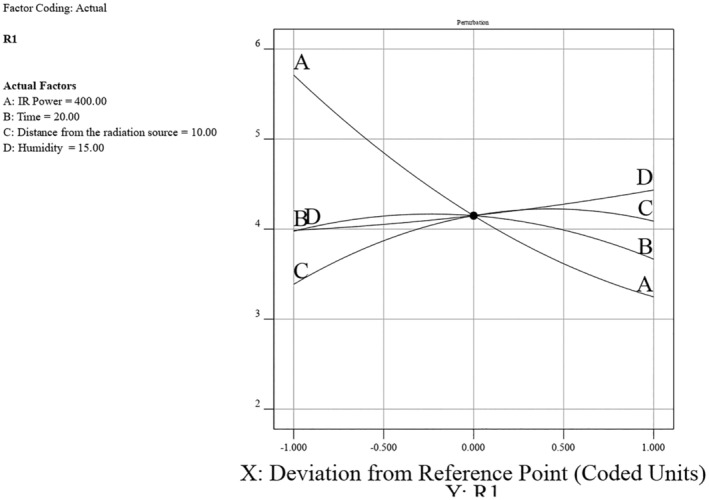
Contour plot of humidity, as a function of two factors: Moisture content (%) and distance from the radiation source (cm).

In this section, factors, including power levels of 300, 400, and 500 W; three time levels of 10, 20, and 30 min; distances from the radiation source of 5, 10, and 15 cm; and sample moisture levels of 10%, 15%, and 20%, were studied. The optimization results indicated that the second‐degree model was significant, suggesting that the selected model was appropriate for the data. Additionally, the *R*‐squared and adjusted *R*‐squared values were 0.9846 and 0.9667, respectively, which are very close to one and indicate the accuracy of the model. Among the factors, the power of the infrared lamp, distance of the lamp, moisture percentage of the sample, and irradiation time were influential, respectively. The contour plot provides a clear visualization of how the factors of initial moisture content and distance from the radiation source interact to influence humidity levels (Figure 1).

**FIGURE 2:**
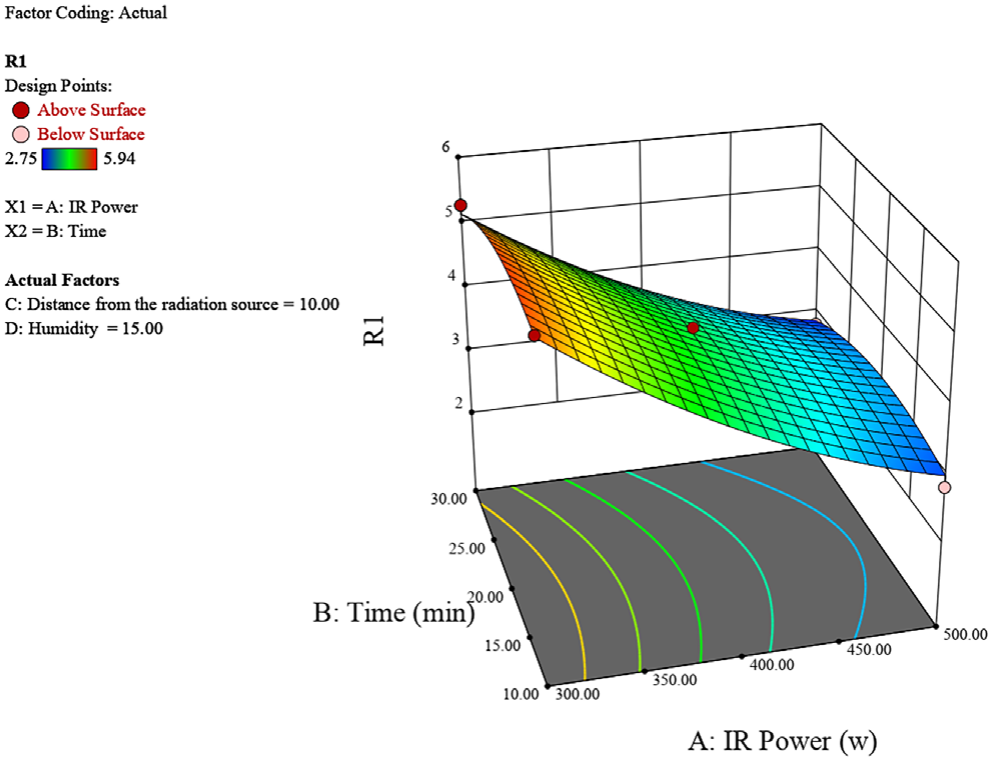
3D surface plot of the effect of radiation power and time on chestnut moisture percentage.

Figure 2 represents how the factors of IR power and irradiation time interact to affect the humidity levels. For optimizing peeling conditions using infrared radiation, higher IR power and longer irradiation time are recommended to achieve higher humidity levels, which are likely advantageous for the peeling process. The highest humidity (around 5.9) is observed at the maximum IR power (500 W) and maximum time (30 min). This region is represented by the red area on the surface plot. Increasing both the IR power and the time leads to a significant increase in humidity. The interaction between IR power and time significantly impacts the humidity levels. As the IR power increases from 300 to 500 W, and the time increases from 10 to 30 min, the humidity also increases consistently.

**FIGURE 3:**
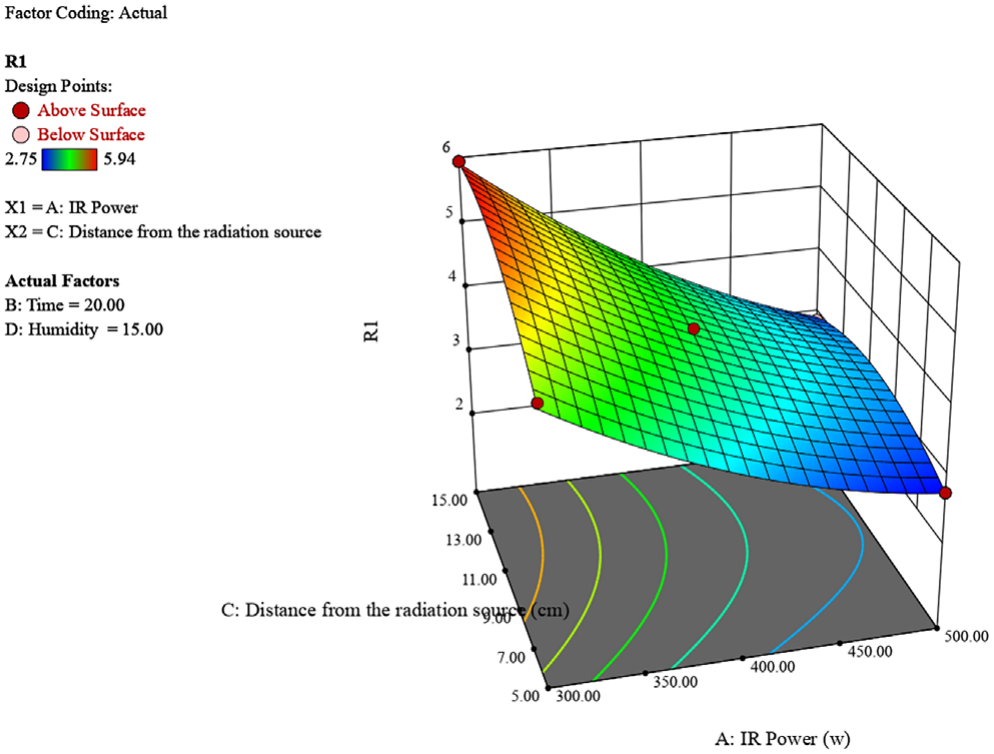
3D surface plot of the effect of radiation power and distance on chestnut moisture percentage.

Figure 3 shows the effect of IR power (300–500 W) and distance from the radiation source (5–15 cm) on humidity (R1). Higher IR power and shorter distances from the radiation source result in higher humidity levels, with the peak humidity around 6 observed at 500 W and 5 cm. Conversely, lower IR power and longer distances lead to lower humidity.

**FIGURE 4:**
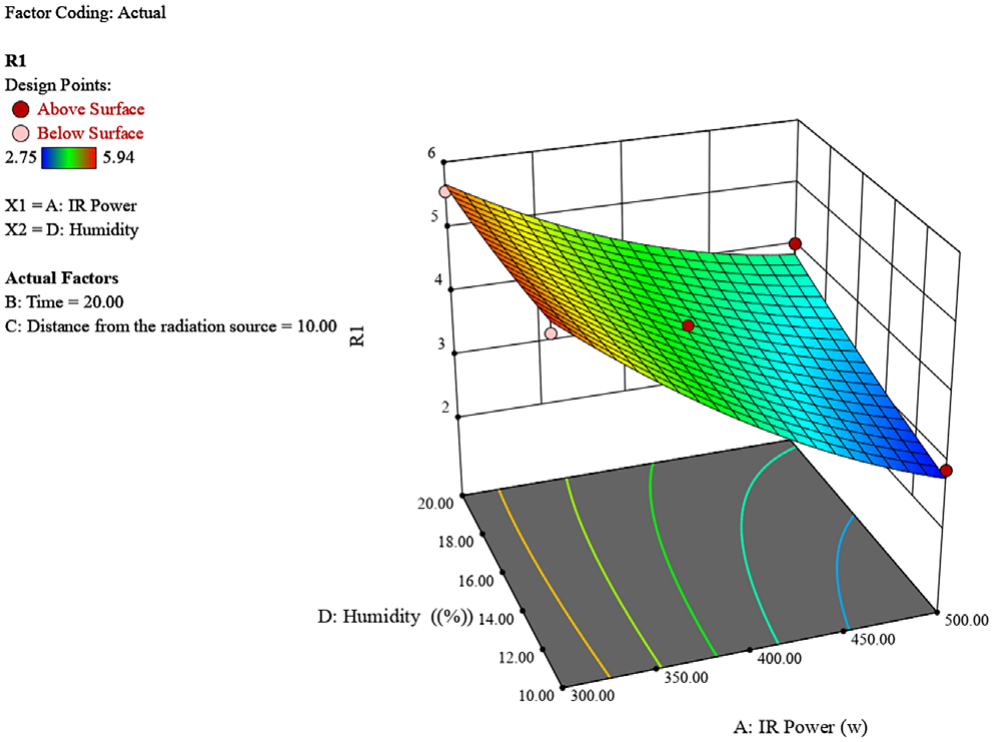
3D surface plot of the effect of radiation power and chestnut moisture percentage on chestnut moisture percentage.

Figure 4 illustrates the impact of IR power (300–500 W) and initial chestnut moisture percentage (10%–20%) on the resulting chestnut moisture percentage. Higher IR power and higher initial moisture percentages yield higher final moisture levels, peaking around 6 at 500 W and 20% moisture. Conversely, lower IR power and moisture percentages result in lower final moisture levels.

**FIGURE 5:**
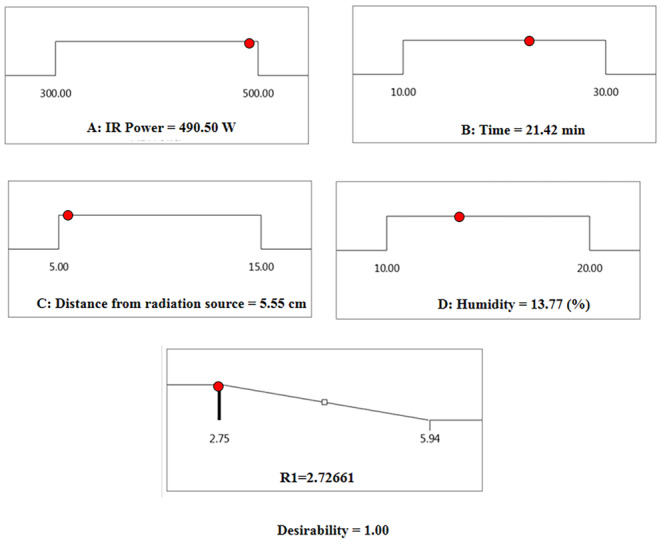
Optimizing the operational conditions of skin peeling with infrared.

The software optimization identified the best conditions for achieving the lowest moisture level at an IR power of 490.50 W, a time of 21.42 min, a radiation distance of 5.55 cm, and an initial moisture content of 13.77%, resulting in a final moisture level of 2.72% (Figure 5).

### 3.2 Optimization of Roasting Conditions Using Infrared Radiation

In the infrared roasting treatment, the factors included power levels of 300, 400, and 500 W; three time levels of 10, 20, and 30 min; distances from the radiation source of 5, 10, and 15 cm; and sample moisture levels of 10%, 15%, and 20%.

**FIGURE 6:**
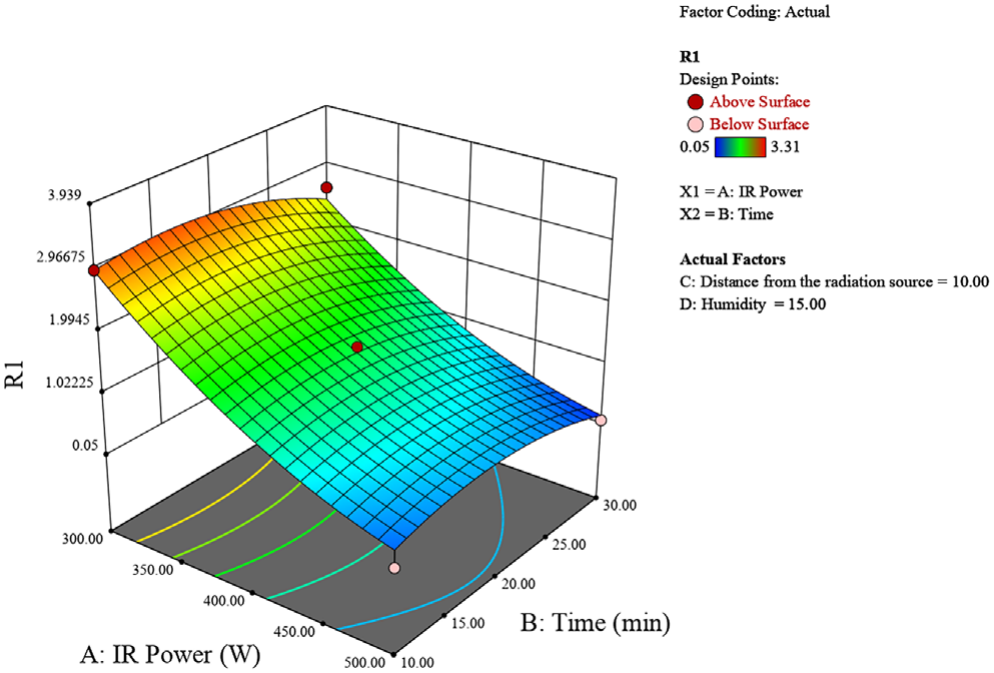
3D surface plot of the effect of radiation power and time on chestnut moisture percentage.

**FIGURE 7:**
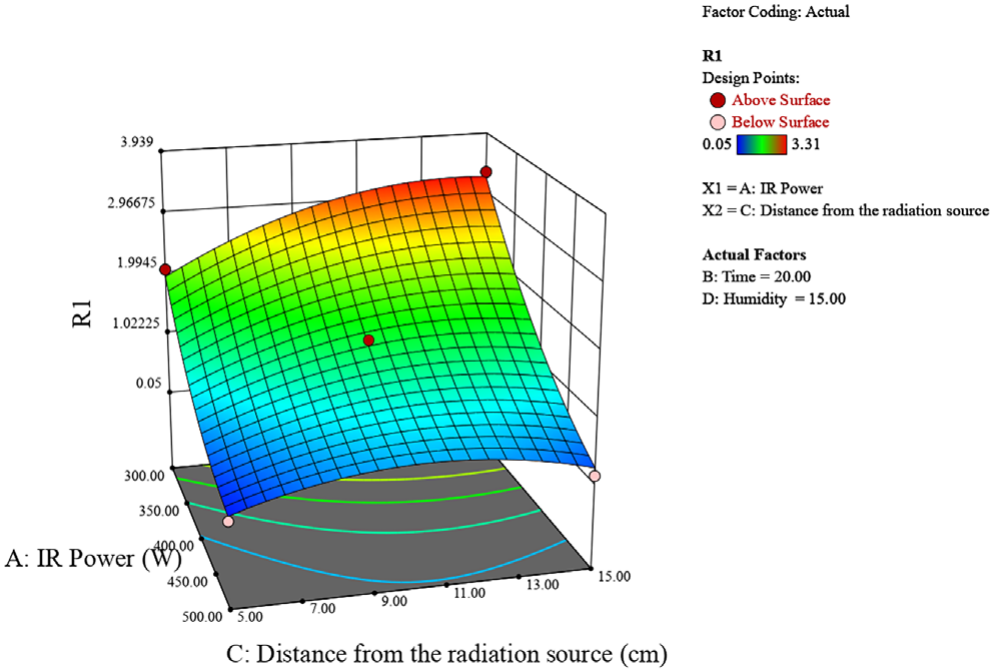
3D surface plot of the effect of radiation power and distance on chestnut moisture percentage.

**FIGURE 8:**
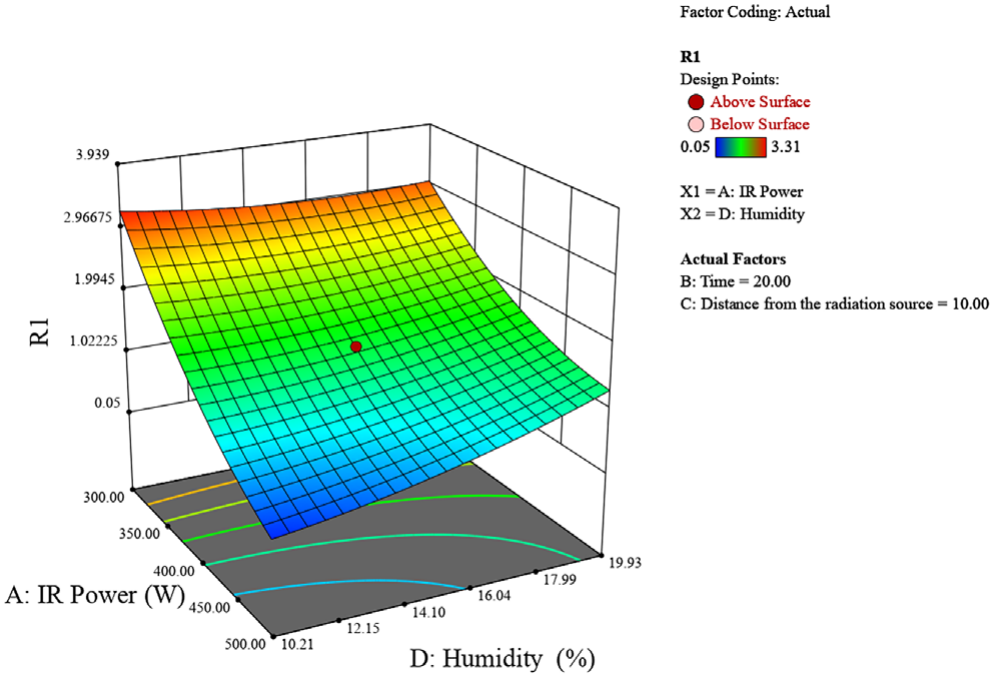
3D surface plot of the effect of radiation power and chestnut moisture percentage on chestnut moisture percentage.

The experimental study on chestnut moisture reduction through infrared (IR) radiation reveals significant insights into the interplay of various factors. The analysis of IR power, irradiation time, distance from the source, and initial moisture percentage shows distinct trends in their effects on chestnut moisture percentage (R). Generally, lower IR powers around 400 W, shorter irradiation times between 10 and 20 min, greater distances of 10–15 cm from the source, and lower initial moisture percentages all contribute to achieving the lowest final chestnut moisture percentages (Figures 6, 7, 8).

**FIGURE 9:**
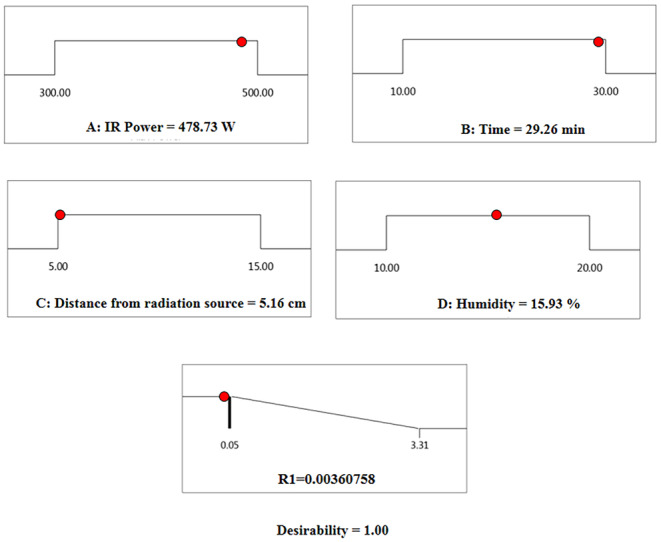
Optimizing the operating conditions of infrared roasting.

The software optimization identified the best conditions for achieving the lowest moisture level at an IR power of 478.73 W, a time of 29.66 min, a radiation distance of 5.16 cm, and an initial moisture content of 15.93%, resulting in a final moisture level of 0.003% (Figure 9).

### 3.3 Results of Chestnut Characteristics

#### 3.3.1 Final Moisture Content

Table 1 presents the results of variance analysis on the final moisture content of chestnuts after different peeling and roasting treatments. As the table shows, the applied treatments significantly affected the moisture content (*p* < 0.05). Looking at the initial moisture content (day 0), manually peeled chestnuts had the highest value, while those roasted with infrared treatment had the lowest. This trend continued after 60 days of storage, with manually peeled chestnuts retaining the highest moisture content and infrared roasted chestnuts having the lowest. Interestingly, the moisture content of roasted chestnuts did not differ significantly between day 1 and day 30 of storage. However, for peeled chestnuts, a significant decrease in moisture content was observed on days 30 and 60.

**TABLE 1:** Final moisture content (% by dry weight) of chestnuts with different treatments.

Treatments	Storage (days)		
	0	30	60
Roasting with optimal infrared conditions	0.94 ± 0.71^Aa^	0.88 ± 0.06^Aa^	0.71 ± 0.27^Aa^
Roasting with optimal hot air conditions	1.59 ± 0.83^Ab^	1.53 ± 0.13^Ab^	1.44 ± 0.02^Ac^
Manual peeling	4.47 ± 1.62^Ad^	3.93 ± 0.79^Bd^	1.62 ± 0.26^Bb^
Infrared peeling	2.80 ± 1.01^Ac^	1.99 ± 1.21^Bc^	1.01 ± 0.01^Bd^

*Note:* Different lowercase letters indicate a significant difference between treatments (*p* < 0.05). Different uppercase letters indicate a significant difference between storage days (*p* < 0.05).

Measuring and assessing the final moisture content is of high importance in roasted kernels, as reactions affecting flavor and taste, such as Maillard reactions, caramelization, and chemical reactions, are directly influenced by moisture. Therefore, this parameter significantly impacts reactions affecting taste and flavor, such as Maillard reactions, caramelization, and chemical reactions during the production process and throughout the storage period after production, thereby having a direct effect on the quality and shelf life of the final product. Roasting leads to gradual heat penetration in the kernel without burning the surface of the grain and, as a result, reduces moisture and increases the crispness of the kernel (Perren and Escher 2013). According to the findings, on all days of storage, the infrared roasting method showed less moisture compared to the hot air roasting method. When food materials are exposed to infrared waves, they heat up quickly, and the moisture gradient (slope) in the food rapidly decreases. During intermittent heating, moisture quickly spreads from the inner layers to the surface, so the final moisture in this method is less than that in the hot air roasting method.

Mokhtari and Ziaiifar (2018) also reported in their study on the effect of different roasting methods on some physicochemical properties of wild almonds that samples roasted with infrared had less final moisture than the hot air method (Mokhtari and Ziaiifar 2018). Furthermore, Bagheri et al. (2016) found similar results in their study on peanut roasting (Bagheri et al. 2016). Additionally, infrared peeling treatments had less final moisture on all days of storage compared to manual peeling treatments, consistent with the results of Oh and Kim (2014). These researchers also reported in their study on differently peeled chestnuts that the moisture content of peeled chestnuts had a significant reduction during a 15‐day storage period in all treatments (Oh and Kim 2014). Vidyarthi et al. (2019) also reported in their study on infrared irradiation peeling of tomatoes that infrared treatment leads to a greater moisture reduction (Vidyarthi et al. 2019).

#### 3.3.2 Texture

Table 2 shows the results of variance analysis on the firmness of chestnuts after different peeling and roasting treatments. The data reveal a significant effect of the treatments on firmness (*p* < 0.05). Initially (day 0), chestnuts with infrared roasting were the least firm, while those with manual peeling were the firmest. Interestingly, firmness increased significantly during storage for all treatments. After 60 days, chestnuts subjected to all treatments exhibited their highest firmness. Looking deeper, hot air roasting resulted in significantly firmer chestnuts compared to infrared roasting. Similarly, manually peeled chestnuts were significantly firmer than those peeled using other methods.

**TABLE 2:** The firmness (N) of chestnuts with different treatments.

Treatments	Storage (days)		
	0	30	60
Roasting with optimal infrared conditions	76.34 ± 1.11^Aa^	87.05 ± 0.63 ^Ba^	101.12 ± 0.28 ^Ca^
Roasting with optimal hot air conditions	85.13 ± 0.68^Ab^	95.88 ± 0.19 ^Bc^	107.26 ± 1.02 ^Cb^
Manual peeling	97.98 ± 4.12^Ac^	106.66 ± 3.01 ^Bd^	136.55 ± 7. 6 ^Cd^
Infrared peeling	88.75 ± 0.87^Ab^	99.18 ± 0.65 ^Bb^	116.75 ± 1.39 ^Cc^

*Note:* Different lowercase letters indicate a significant difference between treatments (*p* < 0.05). Different uppercase letters indicate a significant difference between storage days (*p* < 0.05).

Texture significantly impacts consumer perception of roasted kernel quality (Bagheri et al. 2019). Evaluating texture is, therefore, crucial during new product development and process optimization. Firmness, a key texture attribute, represents the force needed to deform the kernel, either physically or by molars during chewing for solids. Our results align with this concept, showing a decrease in firmness with infrared roasting due to deeper penetration of infrared radiation. Additionally, firmness increased during storage due to gradual moisture loss.

As mentioned, an increase in firmness leads to a decrease in the acceptability of the kernels, making it an important parameter in the characteristics of the kernels. Some studies have examined the effect of roasting on the sensory and textural properties of kernels and the relationship between these two parameters, including studies by Raei et al. (2010) and Shakerardekani et al. (2011) regarding the relationship between sensory and textural properties of pistachio kernels.

#### 3.3.3 Total Phenols

**FIGURE 10:**
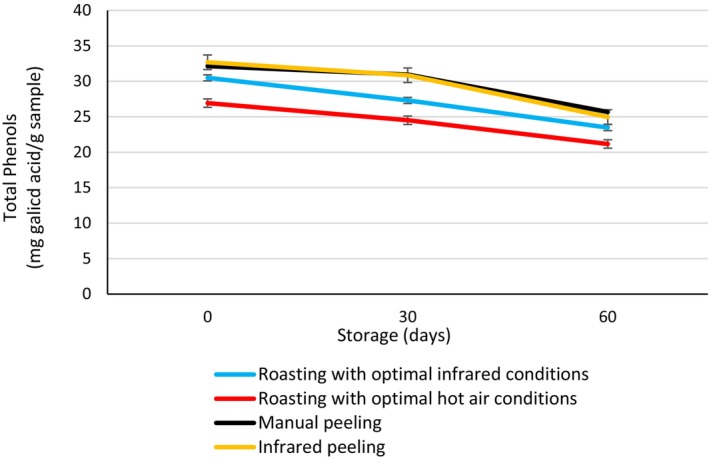
Total phenols of chestnuts with different treatments.

Figure 10 presents the results of variance analysis on the impact of peeling and roasting treatments on total phenol content. Initially (day 0), hot air roasting showed the lowest phenol content, while peeled chestnuts had the highest. Interestingly, total phenols significantly decreased during storage for all treatments, with the lowest levels observed after 60 days. Among roasting treatments, infrared roasting resulted in higher initial phenol content. Notably, no significant difference was observed between peeling treatments. On day 0, chestnut phenolic content ranged from 30 to 32 mg per milliliter (mg/mL) of gallic acid equivalent, indicating a high level of these antioxidant compounds in chestnuts. Phenolic compounds, a diverse group of plant chemicals with unique structures, play a crucial role in antioxidative processes. They scavenge free radicals, deactivate reactive oxygen species (molecules that can damage cells), and chelate metal ions (reducing their harmful effects). These properties contribute to the medicinal, antibacterial, and antioxidant benefits of chestnuts. Their antioxidant effects specifically arise from various mechanisms, including chelating metals, inhibiting free radicals, and influencing enzyme activity (Ham et al. 2015). Thus, examining the level of these compounds in treated chestnuts is very important due to their strong antioxidative properties. The total phenol content of chestnuts in this study was close to the values reported by other researchers. The difference in phenol content depends on the chestnut variety and treatment conditions (Akgün et al. 2021; Martínez et al. 2022).

The results showed that infrared treatment led to an increase in phenolic compounds. The reason for the increase in phenolic compounds is likely due to the enhancement of Maillard browning reactions during roasting. This means that the increase in compounds resulting from browning reactions affects the formation of phenolic compounds, thereby leading to their increase. Compounds participating in the Maillard reaction have phenolic structures, and derivatives from the Maillard reaction include compounds like furans and pyrroles, which may play a role in increasing phenolic compounds in roasted chestnut kernels. The reason for the decrease in phenols during storage is the degradation of phenolic compounds. Bagheri et al. (2016) also reported an increase in phenolic compounds in infrared‐roasted peanuts. Similarly, Vidyarthi et al. (2019) reported an increase in phenolic compounds in tomatoes peeled with infrared.

#### 3.3.4 DPPH

Table 3 presents the results of variance analysis on the impact of peeling and roasting treatments on the DPPH level of chestnuts. The data reveal a significant effect of the treatments on DPPH levels (*p* < 0.05). Interestingly, no significant difference in DPPH levels was observed between treatments on day 0 (*p* > 0.05). However, DPPH levels significantly decreased during storage for all treatments, with the lowest levels observed after 60 days. DPPH (2,2′‐diphenyl‐1‐picrylhydrazyl radical) is one of the oldest methods for indirectly measuring antioxidant properties, based on the reaction of the stable free radical DPPH with hydrogen‐donating compounds such as phenols. The ability of extracts to donate a hydrogen atom to the unpaired DPPH radical is determined by reducing the DPPH radical to its reduced form, DPPH‐H. The purple solution of DPPH changes color to light purple or yellow in reaction with radical inhibitors. The higher the antioxidant power of the compounds, the greater the intensity of the color change (Pinto et al. 2021).

**TABLE 3:** DPPH of chestnuts with different treatments.

Treatments	Storage (days)		
	0	30	60
Roasting with optimal infrared conditions	48.15 ± 0.53^Aa^	44.49 ± 0.90^Ba^	40.81 ± 0.38^Ca^
Roasting with optimal hot air conditions	48.15 ± 1.34^Aa^	44.89 ± 1.02^Ba^	41.42 ± 0.25^Ca^
Manual peeling	48.29 ± 0.57^Ac^	48.04 ± 0.81^Ab^	43.50 ± 0. 2^Bb^
Infrared peeling	48.71 ± 0.09^Aa^	48.00 ± 0.15^Ab^	44.21 ± 0.16^Bb^

*Note:* Different lowercase letters indicate a significant difference between treatments (*p* < 0.05). Different uppercase letters indicate a significant difference between storage days (*p* < 0.05).

Roasting initiates fat oxidation and formation of carbonyl compounds; however, this process, due to the antioxidant effect of products from the Maillard reaction, leads to greater stability of seed oils against oxidation during storage. Oxidative effects of roasting are due to the degradation of natural antioxidants, decomposition of fatty acids, and physical cellular changes. Roasting also alters the oil's breakdown index and flow time, indicating the presence of polymeric substances. Loss of segmentation and increased porosity accelerate mass transfer and thereby facilitate oxygen access to the tissue. Due to the loosening of cell contacts in the nut tissue, the accessible surface area of free cells increases, which, in turn, accelerates the migration of oxygen through the pores via the cell walls into the cells. Therefore, a close relationship between tissue breakdown during roasting and the extent of lipid oxidation can be expected. Lipid oxidation depends much more on the roasting temperature than on the roasting time. The fact that a consistent relationship between roasting conditions and porosity is observed strongly supports the hypothesis that tissue breakdown is the primary factor controlling lipid stability in roasted nuts.

Based on the results, infrared treatments led to an increase in DPPH, which directly correlates with the level of phenolic compounds. An increase in DPPH radical inhibition has been reported in various studies of infrared drying (Osae et al. 2023).

#### 3.3.5 Color Indices

Table 4 presents the results of variance analysis on the effect of peeling and roasting treatments on the lightness index (L*) of chestnuts. As observed in the table, the effect of the treatments was significant at the 5% level (*p* < 0.05). Based on the lightness index results on day 0, the lowest lightness index was associated with manual peeling, while no significant difference was observed among the other treatments. The lightness index of chestnuts showed a significant decrease during storage for all treatments, such that the lowest lightness index for all treatments was after 60 days of storage. The b* index (yellow to blue color scale) of chestnuts demonstrated a significant decrease during storage for all treatments, such that the lowest b* index for all treatments was observed after 60 days of storage. This indicates a decrease in the yellow component of the color, which could be due to changes in the chemical composition of the chestnuts during storage, potentially affecting their appearance and perceived freshness.

**TABLE 4:** Color indices of chestnuts with different treatments after 60 days of storage.

Treatments	Color indices		
	L*	a*	b*
Roasting with optimal infrared conditions	31.19 ± 0.08^b^	41.22 ± 0.38^b^	−3.54 ± 0.87^b^
Roasting with optimal hot air conditions	33.97 ± 0.43^b^	42.97 ± 0.25^b^	−4.76 ± 0.20^b^
Manual peeling	25.85 ± 0.33^a^	21.05 ± 0.20^a^	−2.44 ± 0.65^a^
Infrared peeling	32.84 ± 1.02^b^	43.41 ± 0.26^b^	−1.98 ± 0.87^a^

*Note:* Different lowercase letters indicate a significant difference between treatments (*p* < 0.05).

L*a*b or CIELab is a global standard published in 1967 by the International Commission on Illumination (CIE), where L* represents lightness ranging from 0 to 100, a* represents redness, and b* represents yellowness, both ranging from −120 to +120. Results showed that all treatments significantly demonstrated a reduction in lightness after 60 days of storage (Olatidoye 2021).

The visual color from the consumer's perspective is among the most critical quality characteristics of dehydrated foods. The color is considered an empirical quality index for the process due to the increase in brown pigments as a result of caramelization and browning reactions during roasting (Şakıyan 2015). However, choosing a roasting method based solely on color will lead to flavor defects, as nonenzymatic browning and the resultant color and flavor depend on factors like temperature, water activity, the composition of the food substance, and its pH. The browning reaction rate can be influenced by the amount of reducing sugars present and the size of the product. Samples with fewer reducing sugars or smaller sizes may experience slower browning. This can lead to variations in color development across different sections of the same sample. In smaller samples, localized Maillard reactions may occur, resulting in lighter‐colored areas.

In contrast, roasting promotes nonenzymatic browning, which increases the formation of colored pigments. This generally enhances the visual appeal of the product, contributing to its overall desirability.

Products from the nonenzymatic Maillard and Strecker reactions include various compounds with complex structures, many of which are unsaturated. Double bonds in these structures absorb light, behaving similarly to brown pigments and contributing to the dark color of roasted products. Caramelization of sugar, thermal dehydration, and decomposition of sugars also produce brown pigments, along with organic acids and various aldehydes and ketones (Suleman et al. 2020). Therefore, the reduction in lightness and the increase in browning index in infrared treatments are primarily associated with nonenzymatic browning. Numerous researchers, including Mendes et al. (2001) and Krysiak et al. (2013) in coffee roasting, Kahyaoglu and Kaya (2006) in sesame seed roasting, and Ozdemir and Durs (2000) in hazelnut roasting, have mentioned color changes as one of the main indices for controlling roasting.

Increasing roasting time significantly increased the a* value, likely due to the increased amount of brown pigments from the Maillard reaction, making the samples redder (higher a* index). Similar results were reported by researchers like uysal et al. (2009) and Pourfarzad et al. (2016) where the a* value of samples increased during infrared roasting. Fernando et al. (2014) also showed that the b* value decreases during infrared roasting. Additionally, Pourfarzad et al. (2016) stated that with an increase in infrared radiation power, the b* value first increases and then decreases, suggesting that the increase in b* value is related to the formation of brown pigments during nonenzymatic browning reactions, and its decrease is likely due to the reduction in the L* value.

#### 3.3.6 Browning Index

**FIGURE 11:**
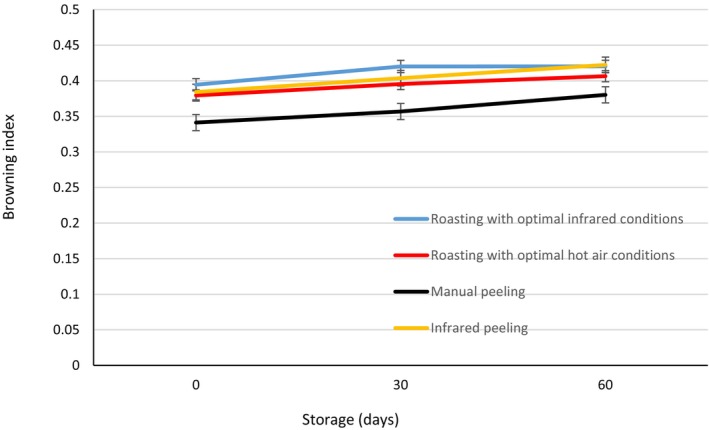
Browning index of chestnuts with different treatments.

Figure 11 presents the results of variance analysis on the browning index of chestnuts after different peeling and roasting treatments. Interestingly, on day 0, manually peeled chestnuts had the lowest browning index, while no significant difference was observed between the other treatments. During storage, the browning index significantly increased for all treatments, with the highest values observed after 60 days. Notably, no significant difference was found between the roasting methods in terms of browning index. Additionally, chestnuts with manual peeling consistently showed a lower browning index compared to other peeling treatments.

#### 3.3.7 Sensory Analysis

Table 5 shows the sensory evaluation results of chestnuts after storage. Manually peeled chestnuts received the lowest scores for color, aroma, and overall acceptance. Conversely, infrared roasting and peeling treatments resulted in the highest scores for color, aroma, and firmness. Interestingly, no significant difference was detected between infrared and hot air roasting in terms of sensory scores.

**TABLE 5:** Sensory evaluation of chestnuts after 60 days of storage.

Treatments	Sensory evaluation				
	Color	Aroma	Roastiness	Firmness	Overall acceptance
Roasting with optimal infrared conditions	4.33 ± 0.65^c^	4.66 ± 0.65^c^	4.57 ± 0.59^a^	4.66 ± 0.65^c^	4.71 ± 0.45^c^
Roasting with optimal hot air conditions	3.76 ± 0.61^b^	4.28 ± 0.61^b^	3.52 ± 0.59^a^	4.28 ± 0.61^b^	4.28 ± 0.77^b^
Manual peeling	3.42 ± 0.59^a^	3.90 ± 0.59^a^	—	3.90 ± 0.59^a^	2.95 ± 1.05^a^
Infrared peeling	4.28 ± 0.70^c^	4.42 ± 0.70^c^	—	4.42 ± 0.70^c^	3.66 ± 1.14^c^

*Note:* Different lowercase letters indicate a significant difference between treatments (*p* < 0.05).

Color or appearance of the kernel is recognized as an important sensory parameter and is used in traditional workshops as a method to determine and measure the degree of roasting. Since this method is nondestructive, quick, and inexpensive, it serves as an indicator for assessing the extent of roasting or the completion of the roasting process (Aboud et al. 2019).

Roasting can improve sensory characteristics. It leads to improvements in the flavor of nut seeds, thereby increasing the desirability of the product. Monosaccharides and amino acids, which are primary flavor precursors, participate in the Maillard reaction and increase the formation of pyrazines. Amino acids and monosaccharides are released from polypeptides and complex carbohydrates through unknown processes during roasting. Among the free amino acids, aspartic acid, glutamic acid, phenylalanine, and histidine are prominent flavor precursors. Sucrose, through inversion to glucose and fructose, participates in the reaction forming flavor and color. Among the products and derivatives from the Maillard reaction, pyrazines are volatile compounds that contribute to the aromatic and roasted smell in food products subjected to high temperatures (Manyatsi et al. 2023). Burnt flavors are typically due to the caramelization reaction in the product. Previous studies have stated that roasting, by altering the internal structure of the kernels, leads to desirable texture creation, making the kernel texture crisp and brittle; hence, the desirability of the texture increases during roasting (Yang et al. 2010; Yang et al. 2013). Overall acceptance is an important parameter for evaluating the effect of roasting on kernels. Indeed, this parameter is used for the final assessment of the roasting effect. Results showed that evaluators gave the lowest score to the manually peeled sample, whereas the infrared roasting treatment received the highest score.

#### 3.3.8 Mold Count

Table 6 highlights the effectiveness of infrared roasting in inhibiting mold growth. both chestnuts with shells and those manually peeled exhibited significant mold growth in the culture medium, while no mold was observed on day 0 for treatments involving peeling and infrared roasting. This trend continued throughout storage; the infrared roasting treatment consistently had the lowest mold count compared to other treatments on days 30 and 60 (Table 6). Infrared radiation can be used to inhibit bacteria, spores, yeasts, and molds in both liquid and solid foods. The effectiveness of infrared inhibition depends on factors such as the amount of infrared energy, food temperature, wavelength, bandwidth, food depth, type of microorganism, moisture content, and the type of food material. Increasing the capacity of the infrared source generates more energy for heating, thereby increasing the total energy absorbed by microorganisms and enhancing microbial inhibition. Various researchers have studied the antimicrobial effect of infrared in different food items, including pistachio kernels (Morshedi and Razavi 2020), peanuts (Golani et al. 2024), wheat grains (Manyatsi et al. 2023), and corn flour (Huang et al. 2021).

**TABLE 6:** Mold count of chestnuts with different treatments.

Treatments	Storage (days)		
	0	30	60
Roasting with optimal infrared conditions	0	5.66 ± 1 ^Aa^	6 ± 0.57 ^Ba^
Roasting with optimal hot air conditions	0	8 ± 1.41 ^Ab^	11.32 ± 2.82 ^Bb^
Manual peeling	0	14 ± 1 ^Ac^	40.33 ± 0. 57 ^Bc^
Infrared peeling	0	8 ± 0.89 ^Ab^	11.33 ± 0.51 ^Ba^

*Note:* Different lowercase letters indicate a significant difference between treatments (*p* < 0.05). Different uppercase letters indicate a significant difference between storage days (*p* < 0.05).

The mechanism of infrared's antimicrobial effect involves thermal inhibition. Infrared radiation damages DNA, RNA, ribosomes, cell membranes, and proteins within microorganisms. Since solid foods have lower thermal conductivity than liquids, infrared heating relies on conductive heat transfer for liquid foods, leading to increased microbial mortality. However, the shallow penetration depth of infrared radiation limits its effectiveness to food surfaces, making it more suitable for surface sterilization.

Results showed that on day 0 of storage, the microbial load of samples was zero, but after 60 days of storage, the infrared treatments had the lowest mold count. These findings align with those of Bingol et al. (2011), who used infrared radiation for the decontamination of almonds. The results indicated that the increase in sample temperature due to the increased power of the infrared lamp was likely the main reason for the further reduction in microbial load during the surface pasteurization of almonds with infrared (Bingol et al. 2011). Additionally, considering the very shallow penetration depth of infrared, an increase in sample thickness is expected to reduce the effectiveness of decontamination by infrared (Coskun et al. 2021).

#### 3.3.9 Electron Microscope Images

**FIGURE 12:**
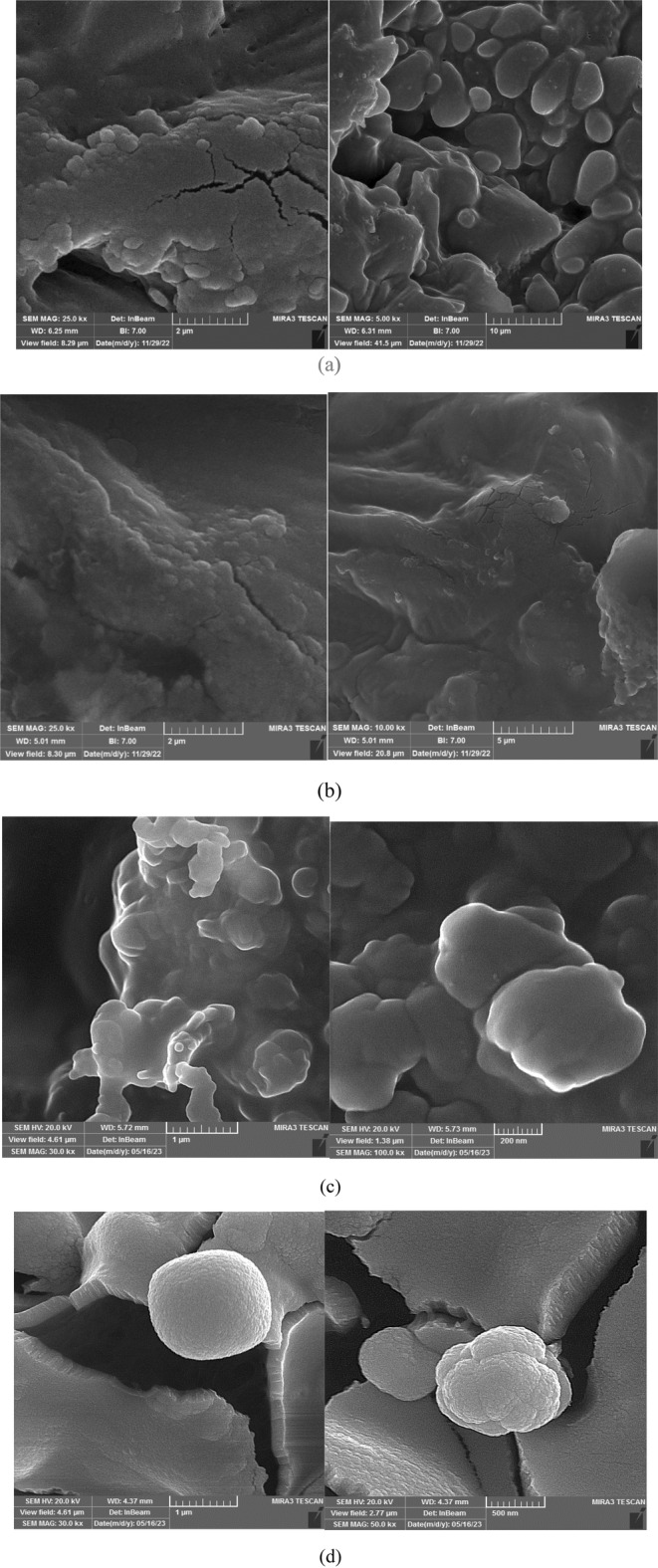
Electron microscope images of chestnuts: (a) Roasted with hot air, (b) Roasted with infrared radiation, (c) Peeled with Infrared Treatment, and (d) Manually peeled.

Figure 12 showcases electron microscope images of chestnuts roasted using hot air (oven) and infrared radiation. The images reveal a striking difference in texture. Chestnuts roasted with infrared exhibit a more uniform structure with greater porosity compared to those roasted with hot air. This increased porosity suggests deeper penetration of heat during infrared roasting, potentially influencing factors such as drying efficiency and flavor development.

## 4 Conclusions

This study showed that infrared radiation can be effectively used for both roasting and peeling chestnuts. By optimizing factors like power, duration, distance, and moisture content, we achieved significant improvements in several areas. Infrared roasting enhanced the physicochemical, antioxidant, and sensory properties of chestnuts while also reducing mold growth during storage. These findings highlight the potential of infrared technology for the food industry. Not only did infrared roasting lead to chestnuts with better shelf life and consumer appeal, but it also paves the way for wider adoption of this technology for similar applications. Overall, this study underscores the importance of infrared treatment as a method for improving food quality and safety, promoting its increased use in food processing and preservation.

## Author Contributions

**Shima Ezzati:** formal analysis (equal), investigation (equal), methodology (equal), software (equal), writing – original draft (equal). **Majid Javanmard dakheli:** conceptualization (equal), data curation (equal), funding acquisition (equal), project administration (lead), supervision (lead), validation (equal), visualization (equal), writing – review and editing (equal). **Hamed Ahari:** conceptualization (equal), data curation (equal), funding acquisition (equal), project administration (equal), supervision (equal), validation (equal), writing – original draft (equal). **Hossein Ahmadi Chenarbon:** data curation (equal), formal analysis (equal), investigation (equal), methodology (equal), validation (equal). **Gholamhassan Asadi:** software (equal), validation (equal), visualization (equal).

## Conflicts of Interest

The authors declare no Conflicts of Interest.

## Data Availability

The data that support the findings of this study are available on request from the corresponding author. The data are not publicly available due to privacy or ethical restrictions.

## References

[fsn370061-bib-0001] Aboud, S. A. , A. B. Altemimi , A. RS Al‐HiIphy , L. Yi‐Chen , and F. Cacciola . 2019. “A Comprehensive Review on Infrared Heating Applications in Food Processing.” Molecules 24, no. 22: 4125. 31731574 10.3390/molecules24224125 PMC6891297

[fsn370061-bib-0002] Akgün, N. , Ö. F. Çelik , and L. Kelebekli . 2021. “Physicochemical Properties, Total Phenolic Content, and Antioxidant Activity of Chestnut, Rhododendron, Acacia and Multifloral Honey.” Journal of Food Measurement and Characterization 15, no. 4: 3501–3508.

[fsn370061-bib-0003] Amiri Chayjan, R. , S. M. T. Bahrabad , and F. Rahimi Sardari . 2014. “Modeling Infrared‐Covective Drying of Pistachio Nuts Under Fixed and Fluidized Bed Conditions.” Journal of Food Processing and Preservation 38, no. 3: 1224–1233.

[fsn370061-bib-0004] Bagheri, H. 2020. “Application of Infrared Heating for Roasting Nuts.” Journal of Food Quality 2020: 1–10.

[fsn370061-bib-0005] Bagheri, H. , M. Kashaninejad , A. M. Ziaiifar , and M. Aalami . 2016. “Novel Hybridized Infrared‐Hot Air Method for Roasting of Peanut Kernels.” Innovative Food Science & Emerging Technologies 37: 106–114.

[fsn370061-bib-0006] Bagheri, H. , M. Kashaninejad , A. M. Ziaiifar , and M. Aalami . 2019. “Textural, Color and Sensory Attributes of Peanut Kernels as Affected by Infrared Roasting Method.” Information Processing in Agriculture 6, no. 2: 255–264.

[fsn370061-bib-0007] Belviso, S. , B. Dal Bello , S. Giacosa , et al. 2017. “Chemical, Mechanical and Sensory Monitoring of Hot Air‐ and Infrared‐Roasted Hazelnuts (*Corylus avellana* L.) During Nine Months of Storage.” Food Chemistry 217: 398–408. 10.1016/j.foodchem.2016.08.103. 27664651

[fsn370061-bib-0008] Bingol, G. , J. Yang , M. T. Brandl , Z. Pan , H. Wang , and T. H. McHugh . 2011. “Infrared Pasteurization of Raw Almonds.” Journal of Food Engineering 104, no. 3: 387–393.

[fsn370061-bib-0009] Buthelezi, N. M. D. , S. Z. Tesfay , K. Ncama , and L. S. Magwaza . 2019. “Destructive and Non‐destructive Techniques Used for Quality Evaluation of Nuts: A Review.” Scientia Horticulturae 247: 138–146.

[fsn370061-bib-0010] Chandrasekara, N. , and F. Shahidi . 2011. “Effect of Roasting on Phenolic Content and Antioxidant Activities of Whole Cashew Nuts, Kernels, and Testa.” Journal of Agricultural and Food Chemistry 59, no. 9: 5006–5014. 21438525 10.1021/jf2000772

[fsn370061-bib-0011] Chung, H.‐S. , J.‐K. Kim , K.‐D. Moon , and K.‐S. Youn . 2014. “Changes in Color Parameters of Corn Kernels During Roasting.” Food Science and Biotechnology 23: 1829–1835.

[fsn370061-bib-0012] Coskun, E. , S. Ozturk , M. Akpinar , A. K. Halkman , and F. Erdogdu . 2021. “Effect of Far Infrared Heating Process on Surface Decontamination and Quality Attributes of Whole Yellow and White Onions.” Food Control 130: 108376.

[fsn370061-bib-0013] Eskandari, J. , A. M. Kermani , S. Kouravand , and P. Zarafshan . 2018. “Design, Fabrication, and Evaluation a Laboratory Dry‐Peeling System for Hazelnut Using Infrared Radiation.” LWT 90: 570–576.

[fsn370061-bib-0014] Fernando, A. , K. Amaratunga , L. Priyadarshana , D. Galahitiyawa , and K. Karunasinghe . 2014. “Roasting Chilli ( *Capsicum annuum* L.) Using Far‐infrared Radiation.”

[fsn370061-bib-0015] Golani, R. , C. Leishangthem , H. Xiao , Q. Zhang , and P. Sutar . 2024. “Effect of High Temperature Short Time Infrared Roasting of Peanuts.” Journal of Future Foods 4, no. 2: 173–178.

[fsn370061-bib-0016] Ham, J.‐S. , H.‐Y. Kim , and S.‐T. Lim . 2015. “Antioxidant and Deodorizing Activities of Phenolic Components in Chestnut Inner Shell Extracts.” Industrial Crops and Products 73: 99–105.

[fsn370061-bib-0017] Huang, D. , P. Yang , X. Tang , L. Luo , and B. Sunden . 2021. “Application of Infrared Radiation in the Drying of Food Products.” Trends in Food Science and Technology 110: 765–777.

[fsn370061-bib-1006] Kahyaoglu, T. , and S. Kaya . 2006. “Modeling of Moisture, Color and Texture Changes in Sesame Seeds During the Conventional Roasting.” Journal of Food Engineering 75, no. 2: 167–177.

[fsn370061-bib-0018] Kate, A. E. , and P. P. Sutar . 2020. “Effluent Free Infrared Radiation Assisted Dry‐Peeling of Ginger Rhizome: A Feasibility and Quality Attributes.” Journal of Food Science 85, no. 2: 432–441. 31968399 10.1111/1750-3841.15009

[fsn370061-bib-1005] Krysiak, W. , R. Adamski , and D. Żyżelewicz . 2013. “Factors Affecting the Color of Roasted Cocoa Bean.” Journal of Food Quality 36, no. 1: 21–31.

[fsn370061-bib-0019] Lamberti, C. , S. Nebbia , S. Antoniazzi , et al. 2021. “Effect of Hot Air and Infrared Roasting on Hazelnut Allergenicity.” Food Chemistry 342: 128174. 33077287 10.1016/j.foodchem.2020.128174

[fsn370061-bib-0020] Lixia, H. , L. Bo , and W. Shaojin . 2015. “Kinetics of Color Degradation of Chestnut Kernel During Thermal Treatment and Storage.” International Journal of Agricultural and Biological Engineering 8, no. 4: 106–115.

[fsn370061-bib-0021] Manyatsi, T. S. , A. R. Al‐Hilphy , M. Majzoobi , A. Farahnaky , and M. Gavahian . 2023. “Effects of Infrared Heating as an Emerging Thermal Technology on Physicochemical Properties of Foods.” Critical Reviews in Food Science and Nutrition 63, no. 24: 6840–6859. 35225100 10.1080/10408398.2022.2043820

[fsn370061-bib-0022] Martínez, S. , C. Fuentes , and J. Carballo . 2022. “Antioxidant Activity, Total Phenolic Content and Total Flavonoid Content in Sweet Chestnut (*Castanea sativa* Mill.) Cultivars Grown in Northwest Spain Under Different Environmental Conditions.” Food 11, no. 21: 3519. 10.3390/foods11213519 PMC9657422 36360132

[fsn370061-bib-1004] Mendes, L. C. , H. C. de Menezes , M. Aparecida , and A. P. Da Silva . 2001. “Optimization of the Roasting of Robusta Coffee (*C. canephora* Conillon) Using Acceptability Tests and RSM.” Food Quality and Preference 12, no. 2: 153–162.

[fsn370061-bib-0023] Mohammadi Moghaddam, T. , S. M. Razavi , M. Taghizadeh , and A. Sazgarnia . 2016. “Sensory and Instrumental Texture Assessment of Roasted Pistachio Nut/Kernel by Partial Least Square (PLS) Regression Analysis: Effect of Roasting Conditions.” Journal of Food Science and Technology 53: 370–380. 26787956 10.1007/s13197-015-2054-2 PMC4711475

[fsn370061-bib-0024] Mokhtari, Z. , and A. M. Ziaiifar . 2018. “The Effect of Different Methods of Roasting on the Physico‐Chemical Properties of Wild Almond.” Innovative Food Technologies 6, no. 1: 55–73.

[fsn370061-bib-0025] Morshedi, A. , and S. M. A. Razavi . 2020. “Effect of Infrared Roasting Process on the Microorganism Contaminations of Long and Round Iranian Pistachio Kernels.” Journal of Nuts 11, no. 1: 23–36.

[fsn370061-bib-0026] Mustafa, A. M. , D. Abouelenein , L. Acquaticci , et al. 2021. “Effect of Roasting, Boiling, and Frying Processing on 29 Polyphenolics and Antioxidant Activity in Seeds and Shells of Sweet Chestnut (*Castanea sativa* Mill.).” Plants 10, no. 10: 2192. 34686001 10.3390/plants10102192 PMC8537430

[fsn370061-bib-0027] Oh, S.‐I. , and M.‐J. Kim . 2014. “Changes in Quality Characteristics of Peeled Chestnut ‘Tsukuba’ According to Storage Temperature and Peeling Method.” Korean Journal of Plant Resources 27, no. 1: 72–79.

[fsn370061-bib-0028] Olatidoye, O. 2021. “Effect of Temperature and Time Combinations on Colour Characteristics, Mineral and Vitamin Content Raw and Roasted Cashew Kernel.” Journal of Food Processing & Technology 12: 554.

[fsn370061-bib-0029] Osae, R. , M. T. Apaliya , E. Kwaw , et al. 2023. “Evaluation of Various Drying Approaches on the Physicochemical Properties, Rehydration Kinetics, Mathematical Modeling and Quality of Tiger Nut (*Cyperus esculentum*).” Journal of Agriculture and Food Research 12: 100584.

[fsn370061-bib-0030] Pan, Z. 2020. “Innovative Infrared Heating Technologies for Food and Agricultural Processing.” Technology and Innovation 21, no. 4: 1–16.

[fsn370061-bib-0031] Perren, R. , and F. Escher . 2013. “Impact of Roasting on Nut Quality.” In Improving the Safety and Quality of Nuts, 173–197. Elsevier.

[fsn370061-bib-0032] Pinto, D. , E. F. Vieira , A. F. Peixoto , et al. 2021. “Optimizing the Extraction of Phenolic Antioxidants From Chestnut Shells by Subcritical Water Extraction Using Response Surface Methodology.” Food Chemistry 334: 127521. 32693333 10.1016/j.foodchem.2020.127521

[fsn370061-bib-0033] Porretta, S. 2019. Tomato Chemistry, Industrial Processing and Product Development. Vol. 9. Royal Society of Chemistry.

[fsn370061-bib-1008] Pourfarzad, A. , S. Gheibi , and Z. Ahmadian . 2016. “Evaluation of Sensory Characteristics and Modeling of the Kinetics of Hazelnut Color Indices During Infrared Roasting.” Innovative Food Technologies 4, no. 2: 81–99.

[fsn370061-bib-0034] Proietti, N. , L. Liguori , V. Di Tullio , G. Adiletta , and P. Russo . 2021. “Investigation on Shelf‐Life of Roasted Chestnuts With Different Packaging Systems Through Low‐Field NMR Analysis.” Chemical Engineering Transactions 87: 97–102.

[fsn370061-bib-0035] Qu, W. , Y. Liu , Y. Feng , and H. Ma . 2022. “Research on Tomato Peeling Using Flame‐Catalytic Infrared Radiation.” LWT 163: 113542.

[fsn370061-bib-1001] Raei, M. , A. Mortazavi , and H. Pourazarang . 2010. “Effects of Packaging Materials, Modified Atmospheric Conditions, and Storage Temperature on Physicochemical Properties of Roasted Pistachio Nut.” Food Analytical Methods 3: 129–132.

[fsn370061-bib-0036] Şakıyan, Ö. 2015. “Optimization of Formulation of Soy‐Cakes Baked in Infrared‐Microwave Combination Oven by Response Surface Methodology.” Journal of Food Science and Technology 52: 2910–2917. 25892790 10.1007/s13197-014-1342-6 PMC4397293

[fsn370061-bib-0037] Schlörmann, W. , M. Birringer , V. Böhm , et al. 2015. “Influence of Roasting Conditions on Health‐Related Compounds in Different Nuts.” Food Chemistry 180: 77–85. 25766804 10.1016/j.foodchem.2015.02.017

[fsn370061-bib-0038] Schmiele, M. 2015. “Physicochemical, Structural and Rheological Properties of Chestnut (*Castanea sativa*) Starch.”

[fsn370061-bib-1002] Shakerardekani, A. , R. Karim , H. M. Ghazali , and N. L. Chin . 2011. “Effect of Roasting Conditions on Hardness, Moisture Content and Colour of Pistachio Kernels.” Moisture Content and Colour of Pistachio Kernels 18: 704–710.

[fsn370061-bib-0039] Shen, Y. , R. Khir , D. Wood , T. H. McHugh , and Z. Pan . 2020. “Pear Peeling Using Infrared Radiation Heating Technology.” Innovative Food Science & Emerging Technologies 65: 102474.

[fsn370061-bib-0040] Siciliano, I. , B. Dal Bello , G. Zeppa , D. Spadaro , and M. L. Gullino . 2017. “Static Hot Air and Infrared Rays Roasting Are Efficient Methods for Aflatoxin Decontamination on Hazelnuts.” Toxins 9, no. 2: 72. 28230792 10.3390/toxins9020072 PMC5331451

[fsn370061-bib-1003] Suleman, R. , T. Hui , Z. Wang , H. Liu , and D. Zhang . 2020. “Comparative Analysis of Charcoal Grilling, Infrared Grilling and Superheated Steam Roasting on the Colour, Textural Quality and Heterocyclic Aromatic Amines of Lamb Patties.” International Journal of Food Science and Technology 55, no. 3: 1057–1068.

[fsn370061-bib-0041] Tu, X. H. , B. f. Wu , Y. Xie , et al. 2021. “A Comprehensive Study of Raw and Roasted Macadamia Nuts: Lipid Profile, Physicochemical, Nutritional, and Sensory Properties.” Food Science & Nutrition 9, no. 3: 1688–1697. 33747479 10.1002/fsn3.2143 PMC7958573

[fsn370061-bib-1007] Uysal, N. , G. Sumnu , and S. Sahin . 2009. “Optimization of Microwave–Infrared Roasting of Hazelnut.” Journal of Food Engineering 90, no. 2: 255–261.

[fsn370061-bib-0042] Vidyarthi, S. , X. Li , and Z. Pan . 2019. “Peeling of Tomatoes Using Infrared Heating Technology.”

[fsn370061-bib-0043] Wang, B. , C. Venkitasamy , F. Zhang , L. Zhao , R. Khir , and Z. Pan . 2016. “Feasibility of Jujube Peeling Using Novel Infrared Radiation Heating Technology.” LWT‐ Food Science and Technology 69: 458–467.

[fsn370061-bib-0044] Wani, I. A. , H. Hamid , A. M. Hamdani , A. Gani , and B. A. Ashwar . 2017. “Physico‐Chemical, Rheological and Antioxidant Properties of Sweet Chestnut (*Castanea Sativa* Mill.) as Affected by Pan and Microwave Roasting.” Journal of Advanced Research 8, no. 4: 399–405. 28649458 10.1016/j.jare.2017.05.005 PMC5470552

[fsn370061-bib-0045] Yang, J. , G. Bingol , Z. Pan , M. T. Brandl , T. H. McHugh , and H. Wang . 2010. “Infrared Heating for Dry‐Roasting and Pasteurization of Almonds.” Journal of Food Engineering 101, no. 3: 273–280.

[fsn370061-bib-0046] Yang, J. , Z. Pan , G. Takeoka , et al. 2013. “Shelf‐Life of Infrared Dry‐Roasted Almonds.” Food Chemistry 138, no. 1: 671–678. 23265539 10.1016/j.foodchem.2012.09.142

[fsn370061-bib-0047] Zhao, X. , X. Ren , H. Liu , X. Zhang , M. Wang , and H. Hu . 2024. “Impact of Steaming and Roasting Heat‐Treatment on Physico‐Chemical and Functional Properties of Walnut Kernel.” Journal of the American Oil Chemists' Society 101, no. 3: 345–360.

